# Supplementary material for: Are open set classification methods effective on large-scale datasets?
Source: PLoS One. 2020 Sep 4;15(9):e0238302. doi: 10.1371/journal.pone.0238302 (PMC7473573; doi:10.1371/journal.pone.0238302)
Supplement: Attachment — Submitted filename: Response_to_Reviewers.pdf [file pone.0238302.s001.pdf]

R·I·T

Rochester Institute of Technology

Ryne Roady
Chester F. Carlson Center for Imaging Science
Rochester Institute of Technology
54 Lomb Memorial Drive
Rochester, NY 14623

Dear Professor Sun and Reviewers,

Thank you for providing a timely response to our journal submission. We believe that your comments and suggestions make this a better article.

The largest issues that we have been asked to address include expanding our analysis. The additional experiments requested by reviewer #1 for analyzing the effect of model capacity to AUROC performance for various inference methods has been included in an updated version of Figure 7. While the additional methods have different levels of open set classification performance as the model capacity is varied the general trends remain the same where AUROC performance generally mirrors the closed set accuracy performance. We do see a different trend for the Mahalanobis distance metric as model width is varied and the inherent dimensionality is increased; however, this method performs significantly worse than other inference methods and is not representative of state-of-the-art OSC performance for large-scale problems. Additional updates have been made in the discussion to improve our analysis of problem difficulty, out-of-distribution similarity, and our analysis of the benefit of feature space regularization toward improving OSC performance.

In response to reviewer #2's request that additional analysis consider independent input perturbation and temperature scaling, we included this analysis in our ablation of the ODIN method and conclude that temperature scaling provides much of the benefit of the ODIN method in large-scale OSC. This is an important finding as temperature scaling requires virtually no additional computation resources during inference and can easily be applied in most settings where a validation data set is available.

Finally, we have ensured that all tables and figures are now properly referred to in the text.

We have combined all of the reviewer responses below along with our changes and comments. Please let us know if you have any further questions or concerns. Thank you for your consideration.

Very Respectfully,
Ryne Roady
PhD Candidate
Rochester Institute of Technology

Responses to Reviewers and Revisions to the Paper

Reviewer #1: While this paper targets large-scale dataset, the studied ResNet only has 18 layers contrast to popular networks that have hundreds of layers and convolutional filters(Lines 433-434). Therefore, the authors further studies the effects of model depth and width in Section 4.4. However, in Figure 7 the authors only consider τ-Softmax and ODIN. Could the authors justify why other inference/regularization methods are not considered?

ODIN was chosen because it was one of the best performing methods, and τ-Softmax was provided as a comparison. We agree that other inference methods can easily be added and serve as additional evidence to our initial analysis that OSC performance generally follows the same trend as closed-set accuracy when the model capacity is varied within a ResNet architecture. We have updated Figure 7 to include the other inference methods in considered in our paper. Additionally we have concluded that including both the Intra-dataset and Inter-dataset data for this ablation is unnecessary as the trends from the inter-dataset (ImageNet-Open) OOD data is the most significant to identifying trends.

Reviewer #1: In Lines 461-463, the authors state that "in general OSC performance decreases as the similarity between the OOD and in-distribution data increases". Could the authors explain this observation?

This was an empirical observation drawn from Figure 5. The underlying reasons for this performance decrease is due to OOD samples from novel classes being confused for known classes. This is likely due to the network learning features to distinguish between classes during normal discriminative training; however, if an OOD image shares some of these distinguishing features with a known class then there is a higher likelihood that the OOD image will be incorrectly identified.

We added this hypothesis in the discussion section following the discussion of feature space regularization strategies:

Fundamentally, the increase in OSC difficulty as the similarity increases between OOD and in-distribution samples is due to the network confusing OOD inputs with known classes. This confusion stems from the feature space of the CNN classifier which learns to be most sensitive to variations in the training distribution that are semantically meaningful while ignoring variations that are not semantically meaningful among the known classes. Dealing with semantically meaningful variations in images from both known and unknown classes that are not included in the training set is ultimately the most significant problem in the OSC process.

Reviewer #1: In Lines 428-431, the authors says the resulting ROC curves "demonstrate that there is little to no benefit from background class regularization versus standard cross-entropy training in the open set classification task". However, in Table 2 it seems that the AUOSC values from background class regularization in ImageNet-Open do have some improvement versus the AUOSC values in cross-entropy. Additionally, in previous section the authors mention that AUOSC metric is a better indicator when comparing regularization techniques.

Therefore, I feel that the authors should make a more comprehensive conclusion by considering Table 2 and Figure 6 together.

We have tempered this statement in describing the lack of benefit from background regularization in large-scale OSC problems. We have added the additional observation that while the ROC charts qualitatively appear to show little benefit from the background class regularization, we have nevertheless shown statistically significant increases in AUROC performance from this feature space regularization approach. The specific wording of the paragraph has been changed to:

In Fig. 6 we also show the resulting ROC curves for the ImageNet Intra-Dataset problem across the three feature spaces tested. While qualitatively there appears to be little benefit from background class regularization versus standard cross-entropy training we did find significant differences in the AUROC metric calculated across the full range of OOD detection thresholds as reported in Table 2.

Reviewer #1: *In Figure 7, it seems that the legends don't tell which curves are from Intra-Dataset.*

We have updated Figure 7. It now includes a wider variety of inference methods as explained in the first response above.

Reviewer #2: I don't know if it is the problem with the latex template but why all figures are at the bottom of the paper? Please reorganize it if it is not the template matter.

The submission template for PLOS ONE required figures to be submitted separately from text.

Reviewer #2: I think the authors should consider to add more details on ablation study to this work, considering independent input perturbation and temperature scaling factors are all well studied in different works. Input perturbation is essentially a data augmentation method that has been widely used in improving DNN performances. The authors of the paper should considering more data augmentation methods in their approach and evaluate which one is better.

We have included in our ablation of the ODIN method an independent analysis of input perturbation and temperature scaling. While we believe different data augmentation methods will have a significant effect on OOD detection performance, we focused on using the standard methods used by the creators of the approaches we are comparing to assess their capabilities with different inference methods on large-scale datasets.
